# Leber’s hereditary optic neuropathy following unilateral painful optic neuritis: a case report

**DOI:** 10.1186/s12886-020-01461-6

**Published:** 2020-05-18

**Authors:** Chaeyeon Lee, Kyung-Ah Park, Ga-In Lee, Sei Yeul Oh, Ju-Hong Min, Byoung Joon Kim

**Affiliations:** 1 Department of Ophthalmology, Samsung Medical Center, Sungkyunkwan University School of Medicine, 81 Irwon-ro, Gangnam-gu, Seoul, 06351 South Korea; 2 Department of Neurology, Samsung Medical Center, Sungkyunkwan University School of Medicine, Seoul, South Korea

**Keywords:** Leber’s hereditary optic neuropathy, Painful, Optic neuritis

## Abstract

**Background:**

Leber’s hereditary optic neuropathy (LHON) is a maternally inherited mitochondrial disease, characterized by acute or subacute, painless, bilateral visual loss. LHON is often misdiagnosed as optic neuritis at an early stage because of the similarity of their clinical presentation. To date, there has been no reported case of actual optic neuritis and LHON in one patient.

**Case presentation:**

A 40-year-old, healthy man was referred to our clinic with acute painful visual loss in the right eye for 2 weeks. In the right eye, visual acuity decreased to 20/40, and the Ishihara colour test score was 8/14 with a relative afferent pupillary defect. Optic disc swelling was found only in the right eye, and magnetic resonance imaging revealed enhancement of the the right optic nerve, consistent with optic neuritis. After receiving 1 g of intravenous methylprednisolone daily for three days, his ocular pain resolved, and visual acuity improved to 20/20 within 2 weeks. Seven months later, the patient developed acute painless visual loss in the right eye. Visual acuity decreased to 20/200 in the right eye. There was no response to the intravenous methylprednisolone therapy at that time. Eight months later, he developed subacute painless visual loss in the left eye. Genetic testing for LHON was performed and revealed the pathologic mtDNA 11778 point mutation.

**Conclusions:**

We report a case with painful unilateral optic neuritis preceding the onset of LHON. Even if a typical optic neuritis patient has completely recovered from steroid treatment once in the past, it is advisable to keep in mind the possibility of LHON if acute or subacute loss of vision subsequently or simultaneously occurs in both eyes and does not respond to steroids.

## Background

Leber’s hereditary optic neuropathy (LHON) is a maternally inherited mitochondrial disease, characterized by acute or subacute, painless, sequentially or simultaneously bilateral visual loss [1]. LHON is often misdiagnosed as optic neuritis at an early stage because of the acute nature of the disease that can develop in one eye [2–4]. To date, there has been no reported case of actual optic neuritis and LHON in one patient. Herein, we report a rare case with painful monocular optic neuritis and complete visual recovery after steroid treatment, preceding the onset of LHON in both eyes.

## Case presentation

**Fig. 1:**
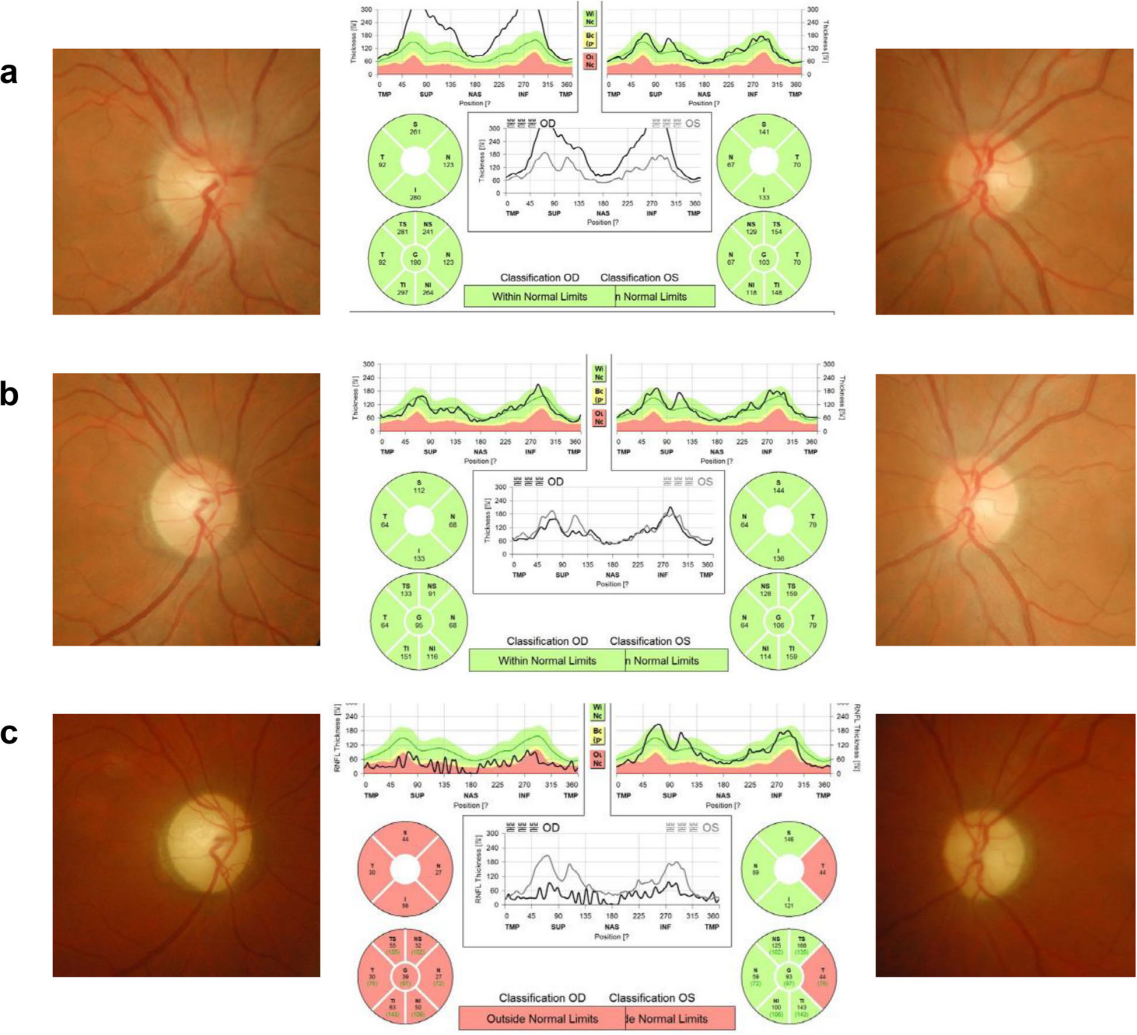
Fundus photography and optical coherence tomography results at each episode. **a,** At the first episode of optic neuritis in the right eye (acute optic neuritis, OD), there was optic disc swelling in the right eye with no abnormality in the left eye. **b,** Seven months after the right optic neuritis, the patient developed acute painless visual loss in the right eye (LHON, OD). There was no optic disc swelling in the right eye with mild temporal disc pallor. **c**, 22 months after the right optic neuritis and 4 months after the new development of left optic neuropathy (4 months after bilateral development of LHON), there was global disc pallor in the right eye and temporal disc pallor in the left eye

**Fig. 2:**
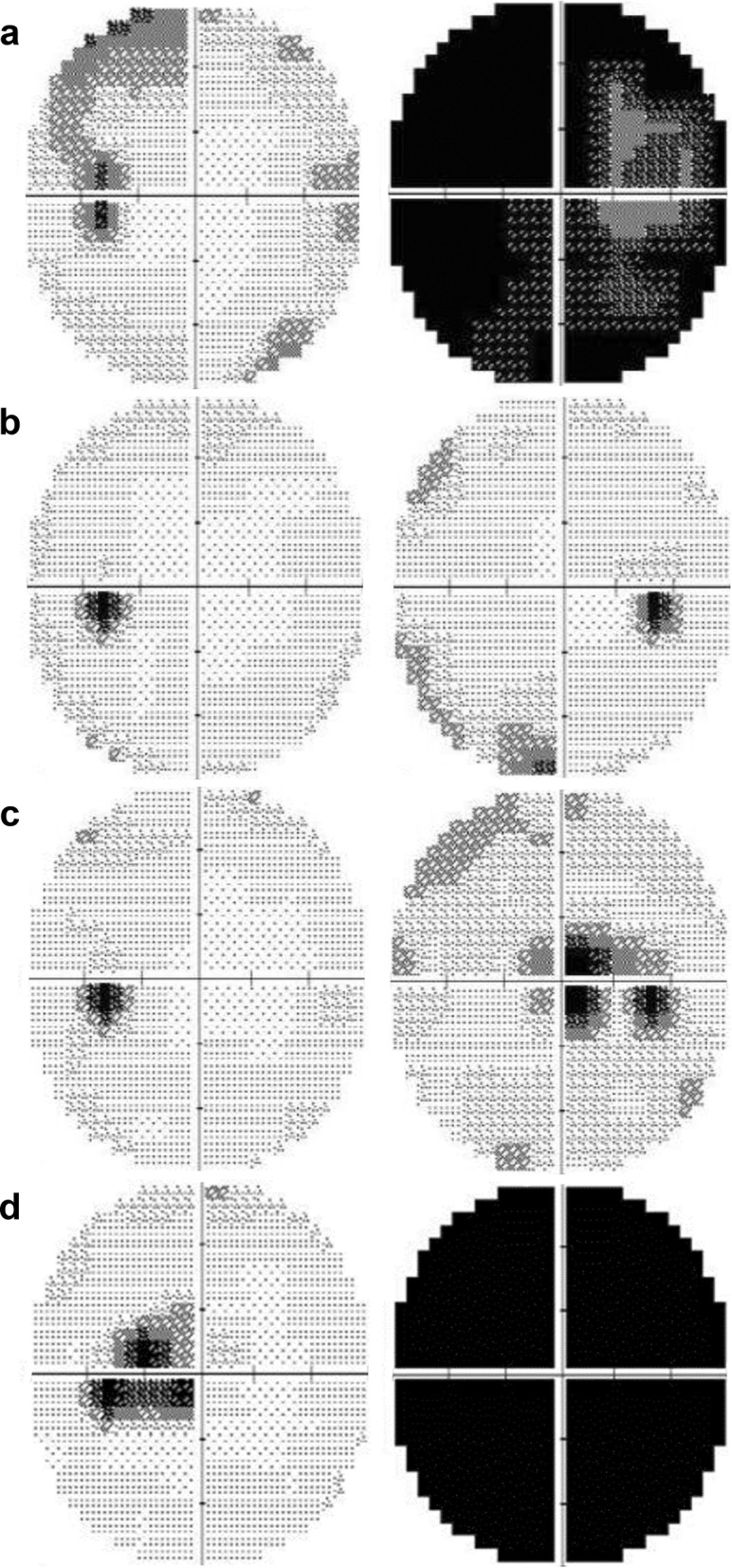
Visual field test results at each episode. **a**, At the first episode of right optic neuritis (acute optic neuritis, OD), there were global visual field defects in the right eye and a superotemporal visual field defect in the left eye. **b**, Five months after the right optic neuritis, there were recoveries of visual field defects in both eyes. **c**, Seven months after the right optic neuritis, the patient developed acute painless visual loss in the right eye (LHON, OD). Central scotoma developed in the right eye. **d**, 18 months after the right optic neuritis, visual loss developed in the left eye (LHON, OS). There were global field defects in the right eye and central scotoma in the left eye

**Fig. 3:**
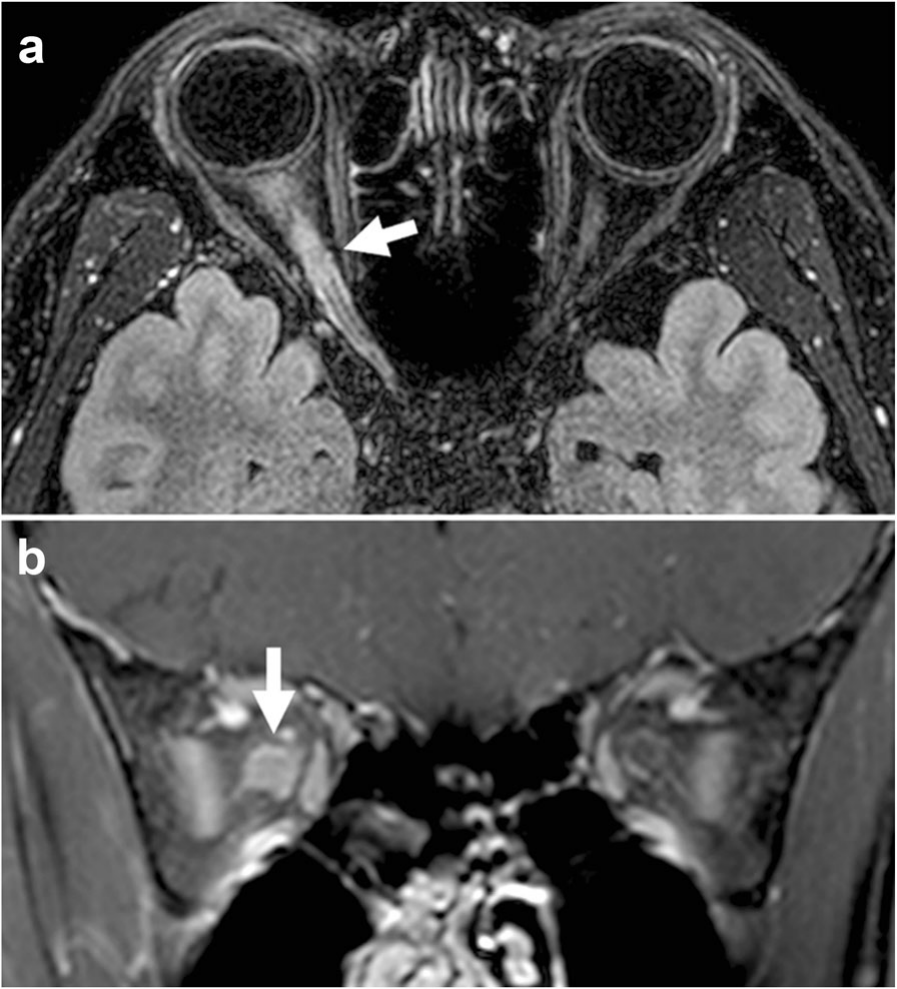
Contrast-enhanced three-dimensional fluid-attenuated inversion recovery-volume isotopic turbo spin echo acquisition (FLAIR-VISTA) axial image with fat suppression (**a**) and a contrast-enhanced T1-weighted coronal image with fat suppression (**b**) showing enhancement of the right optic nerve (arrows)

A 40-year-old, healthy man was referred to our clinic with acute painful visual loss in the right eye for 2 weeks. He suddenly developed acute blurriness in the right eye 2 weeks prior. He developed retrobulbar pain at the same time, which worsened on ocular movement. Over the course of 2 weeks, the blurriness in the right eye worsened. He did not experience any visual discomfort before the recent onset of unilateral vision loss. The patient had no significant social, past medical, past surgical, or trauma history. There was no current medication. He had one maternal uncle who was nearly blind in both eyes (unknown cause) and passed away. He also had a nephew (son of his younger brother) who had strabismus and decreased vision in one eye. A review of systems revealed no other symptoms except progressive visual loss with ocular pain. Upon ocular examination, visual acuity registered 20/40 in the right eye and 20/20 in the left eye. The Ishihara colour test score was 8/14 in the right eye and 12/14 in the left eye. The right pupil reacted slowly and weakly and the left pupil reacted normally to direct light. There was a relative afferent pupillary defect in the right eye. The ocular motility examination was normal. The anterior segments were normal. On fundus examination, there was optic disc swelling in the right eye with no abnormality in the left eye (Fig. 1-a). A visual field test showed there was a generalized visual field defect in the right eye and superotemporal visual field defect in the left eye (Fig. 2-a). Magnetic resonance imaging (MRI) revealed definite enhancement of the right optic nerve, consistent with optic neuritis. The optic nerve of the left eye showed normal MRI findings from the optic nerve head to the chiasm. No periventricular white matter lesion or brain parenchymal lesion was found (Fig. 3). He was given 1 g of intravenous methylprednisolone daily for three days. His ocular pain resolved, and visual acuity improved to 20/20 within 2 weeks after the steroid pulse treatment (Fig. 2-b).

Seven months later, the patient developed acute painless visual loss in the right eye. Visual acuity decreased to 20/200 in the right eye. He showed a central scotoma in the right eye, while visual acuity and the visual field in the left eye were normal (Fig. 2-c). On fundus examination, there was no optic disc swelling in the right eye, with mild temporal disc pallor. No macular or vascular change was observed (Fig. 1-b). MRI showed no enhancement in either optic nerve. The patient was given 1 g of intravenous methylprednisolone daily for three days again, but his visual acuity did not improve.

Eight months later, the patient developed subacute painless visual loss in the left eye. The visual acuity in the left eye deceased to 2/100 with central scotoma in the left eye (Fig. 2-d). MRI showed no enhancement in either optic nerve. Genetic testing for LHON was performed and revealed the pathologic mtDNA 11778 point mutation.

Four years later since onset of bilateral visual loss, this patient’s final visual acuity was registered as 20/500 in the right eye and 20/667 in the left eye. On fundus examination, optic disc pallor was observed in both eyes.

At each episode, neuromyelitis optica immunoglobulin G was performed and every result was negative.

## Discussion and conclusions

LHON is a mitochondrial disease caused by a genetic mutation [1]. It is reported that it can be misdiagnosed in its early stage as other types of optic neuropathies [2–5]. Discrimination between LHON and optic neuritis is very important clinically because of associated acute vision loss. Our case initially presented with typical symptoms and signs of optic neuritis. Unlike in LHON, the patient complained of significant ocular pain, and the pain was exacerbated by eye movement. Our patient also demonstrated definite enhancement of the optic nerve on MRI. Optic nerve enhancement in LHON has been reported in some cases [6–8], but it is known to be rare [9]. When considering the pain relieved by steroid therapy and complete visual improvement, it can be assumed that the initial visual loss of the patient was caused by optic neuritis rather than LHON.

In this case, it is not clear whether optic neuritis and LHON were coincidental findings or whether there was an underlying pathogenic relationship. Previously, various conditions related to the typical types of optic neuropathies such as trauma [10], nutritional deficit [11], and ethambutol administration [12] were suggested to be precipitating factors for developing LHON. In our case, it is possible that optic neuritis acted as a factor precipitating the onset of LHON. A subset of LHON patients who presented with multiple sclerosis-like features has been reported in many past studies [13–19]. Histopathologic results of central nervous tissue in these patients were consistent with inflammatory demyelinating multiple sclerosis-like plaques [18]. Considering optic neuritis is a common initial manifestation of multiple sclerosis, it is also possible that optic neuritis is associated with LHON, similar to a subset of LHON with multiple sclerosis-like features. Even if we assume that optic neuritis was associated with LHON in this case, it is still unclear whether optic neuritis acted as a triggering factor for the onset of LHON or certain mechanisms were shared by these two diseases. We hope that future studies clearly reveal the underlying mechanisms related to these two diseases.

One of the limitations in our study is the lack of antibody testing for myelin oligodendrocyte glycoprotein, which is known to be related to recurrence of optic neuritis [20].

We reported a case with painful unilateral optic neuritis preceding the onset of LHON. Even if a typical optic neuritis patient has completely recovered from steroid treatment once in the past, it is advisable to keep in mind the possibility of LHON if acute or subacute loss of vision subsequently or simultaneously occurs in both eyes and does not respond to steroids.

## Data Availability

All data supporting the findings are contained within the manuscript.

## Abbreviations

LHON: Leber’s hereditary optic neuropathy
MRI: Magnetic resonance imaging
